# Geographic disparities and temporal changes of COVID-19 hospitalization risks in North Dakota

**DOI:** 10.3389/fpubh.2023.1062177

**Published:** 2023-03-16

**Authors:** Md Marufuzzaman Khan, Nirmalendu Deb Nath, Matthew Schmidt, Grace Njau, Agricola Odoi

**Affiliations:** ^1^Department of Public Health, College of Education, Health, and Human Sciences, University of Tennessee, Knoxville, TN, United States; ^2^Department of Biomedical and Diagnostic Sciences, College of Veterinary Medicine, University of Tennessee, Knoxville, TN, United States; ^3^North Dakota Department of Health and Human Services, Special Projects and Health Analytics, Bismarck, ND, United States

**Keywords:** COVID-19, North Dakota, spatial epidemiology, geographic disparities, FlexScan, SaTScan, Geographic Information Systems

## Abstract

**Background:**

Although the burden of the coronavirus disease 2019 (COVID-19) has been different across communities in the US, little is known about the disparities in COVID-19 burden in North Dakota (ND) and yet this information is important for guiding planning and provision of health services. Therefore, the objective of this study was to identify geographic disparities of COVID-19 hospitalization risks in ND.

**Methods:**

Data on COVID-19 hospitalizations from March 2020 to September 2021 were obtained from the ND Department of Health. Monthly hospitalization risks were computed and temporal changes in hospitalization risks were assessed graphically. County-level age-adjusted and spatial empirical Bayes (SEB) smoothed hospitalization risks were computed. Geographic distributions of both unsmoothed and smoothed hospitalization risks were visualized using choropleth maps. Clusters of counties with high hospitalization risks were identified using Kulldorff's circular and Tango's flexible spatial scan statistics and displayed on maps.

**Results:**

There was a total of 4,938 COVID-19 hospitalizations during the study period. Overall, hospitalization risks were relatively stable from January to July and spiked in the fall. The highest COVID-19 hospitalization risk was observed in November 2020 (153 hospitalizations per 100,000 persons) while the lowest was in March 2020 (4 hospitalizations per 100,000 persons). Counties in the western and central parts of the state tended to have consistently high age-adjusted hospitalization risks, while low age-adjusted hospitalization risks were observed in the east. Significant high hospitalization risk clusters were identified in the north-west and south-central parts of the state.

**Conclusions:**

The findings confirm that geographic disparities in COVID-19 hospitalization risks exist in ND. Specific attention is required to address counties with high hospitalization risks, especially those located in the north-west and south-central parts of ND. Future studies will investigate determinants of the identified disparities in hospitalization risks.

## 1. Introduction

The first COVID-19 case in the US was detected in January 2020, and as of August 30, 2022, the US has reported the highest number of confirmed cases globally (94,110,810), with 1,039,055 deaths (1, 2). The disease was reported in every US state although its incidence and severity varied geographically. This might be due to geographic differences in behavioral and demographic factors such as smoking history, co-morbidities, and environmental pollutants which have been shown to play a role in COVID-19 incidence, hospitalization, and mortality risks (3–6). Geographic differences in vaccination rates are another factor that might have impacted geographic disparities in hospitalization rates. According to a report published by the Centers for Disease Control and Prevention (CDC), COVID-19 hospitalization rates were 29.2 times higher in unvaccinated individuals than those who were fully vaccinated (7). National surveillance data demonstrated that overall hospitalization rates increased in the US due to the emergence of COVID-19 (1, 8). Therefore, regular monitoring of hospitalization rates, clinical features, and disposition of hospitalized patients is essential to understanding the epidemiology of COVID-19 in the US, and is also helpful for guiding, planning and prioritizing healthcare resource utilization.

Just like the other US states, North Dakota, a Midwest US state sharing borders with Canada, was affected by the COVID-19 pandemic directly, as well as the disease's health, social and economic ramifications. Unfortunately, little is known about the disparities in COVID-19 burden in North Dakota. A study conducted among the North Dakota tribal people of Spirit Lake reported incidence rates of 520–600 cases per 100,000/person-week, which was 1.5 times higher than the state average during the same time period (9). Knowledge of the burden and geographic disparities of COVID-19 risks in North Dakota is important in guiding the planning and provision of health services and ensuring that the healthcare system is not overwhelmed by high numbers of patients in some geographic locations. Therefore, the objective of this study was to identify geographic disparities of COVID-19 hospitalization risks in North Dakota. Two statistically rigorous approaches are used to identify statistically significant clusters of hospitalization risks. Geographic distribution of identified clusters are presented in maps.

## 2. Methods

### 2.1. Study area and data sources

**Figure 1:**
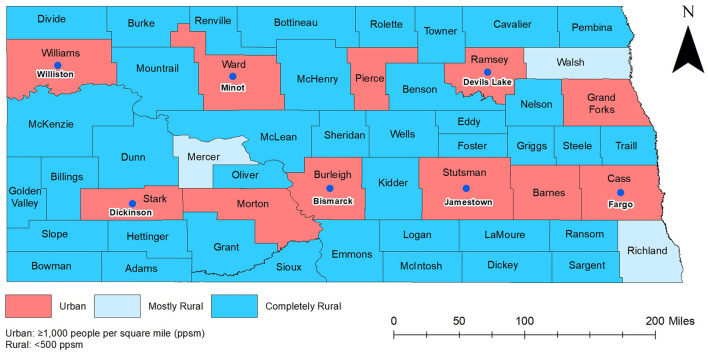
Map of North Dakota showing geographic distribution of urban and rural counties.

The study area encompassed the entire state of North Dakota (Figure 1). As of 2020, North Dakota had the fifth lowest population among all states of the United States of America and had a population of ~0.8 million. The state has 53 counties of which 38 have population densities lower than seven persons per square mile (10, 11). Cass county is the most populous (179,937 residents), while Slope county is the least populous with only 788 residents. The age distribution of the population is 0–19 years old (26.2%), 20–44 years old (35.4%), 45–64 years old (23%), and ≥65 years old (15.3%). The overall racial composition of North Dakota is ~86.9% White, 5.6% Native American, 3.4% Black, and all other races comprise 4.1% of the population. By ethnicity, the majority (95.9%) of the population is non-Hispanic (12).

Data on COVID-19 hospitalized cases covering the time period from March 2020 to September 2021 was collected by the North Dakota Department of Health and Human Services (NDHHS) through COVID-19 case interviews and hospital reporting. A COVID-19 hospitalized case was defined as a person who had a first-time diagnosis of COVID-19 confirmed by laboratory tests and was admitted to a hospital. Only first-time diagnosed and hospitalized cases were included in the study because re-infected cases have a lower risk of hospitalization compared to first-time infections (13). Additional case data provided by HHS included county of residence and age. Data on county-level total population and population of different age categories were extracted from the 2016–2020 American Community Survey 5-years average estimate (14). Cartographic boundary file for county-level geographic analysis was downloaded from the United States Census Bureau TIGER Geodatabase (15). Data on urban and rural classification were obtained from the US Census Bureau (16).

### 2.2. Data preparation and visualization

All descriptive statistical analyses were performed in SAS 9.4 (17). Age was categorized into ≤ 19 years, 20–44 years, 45–64 years, and 65 years or older. Monthly hospitalization risks were computed and expressed as the number of hospitalized cases per 100,000 persons. The study period was divided into peak and non-peak periods as follows: if there were ≥25 hospitalized cases per 100,000 persons in a month, it was identified as a month of the peak period, otherwise it was classified as a non-peak period. Two peak and two non-peak periods were identified. The first peak period started in August 2020 and lasted through December 2020, and the second peak period spanned from August 2021 to September 2021. The first and second non-peak periods were March 2020 to July 2020 and January 2021 to July 2021, respectively. Temporal changes in monthly COVID-19 hospitalized cases per 100,000 persons were assessed graphically in Microsoft Excel 2022 (18). Difference between average hospitalization risks in peak and non-peak periods were compared using two sample *t*-Test.

For each of the four time periods, COVID-19 hospitalized cases were aggregated to the county level. Direct age-adjusted county-level hospitalization risks were calculated using the county-level total population as the denominator and the 2010 US population as the standard (19). Spatial Empirical Bayes (SEB) smoothed age-adjusted hospitalization risks were then calculated in GeoDa 1.14 (20) using 1^st^ order Queen contiguity spatial weight.

Based on the US Census Bureau definition, North Dakota counties were classified into the following three categoris: (1) Urban (<50% of county population live in rural areas); (2) Mostly rural (50–99.9% of county population live in rural areas); (3) Completely rural (100% of county population live in rural areas) (Figure 1) (13).

### 2.3. Detection of clusters

#### 2.3.1. Kulldorff's circular spatial scan statistics

Kulldorff's circular spatial scan statistics (CSSS), implemented in SaTScan 9.6 (21), was used to identify circular clusters of age-adjusted high hospitalization risks. A discrete Poisson probability model specifying circular non-overlapping purely spatial high-risk clusters was used. The maximum circular window size was set at 24% of the population at risk based on the population of Cass County, which has the largest population in North Dakota. This ensures that all counties have a chance of being included in a cluster regardless of their population size. To identify statistically significant clusters, 999 Monte Carlo replications and likelihood ratio test were used specifying a critical *p*-value of 0.05.

#### 2.3.2. Tango's flexible spatial scan statistics

Both circular and irregularly shaped high-risk clusters of age-adjusted hospitalization risks were investigated using Tango's flexible spatial scan statistics (FSSS) implemented in FleXScan 3.1.2 (22, 23). Poisson probability models with a restricted log likelihood (LLR) ratio (specifying an alpha of 0.2) and a maximum cluster size of 15 counties were specified to preclude potential inclusion of counties with non-elevated hospitalization risks. For statistical inference, 999 Monte Carlo replications were used, and statistical significance was assessed using a critical *p*-value of 0.05.

### 2.4. Cartographic displays

QGIS (24) was used to display the geographic distribution of both smoothed and non-smoothed COVID-19 hospitalization risks and the location of spatial clusters. Jenk's optimization classification scheme was used to determine critical intervals for choropleth maps.

## 3. Results

### 3.1. Spatial and temporal patterns

**Figure 2:**
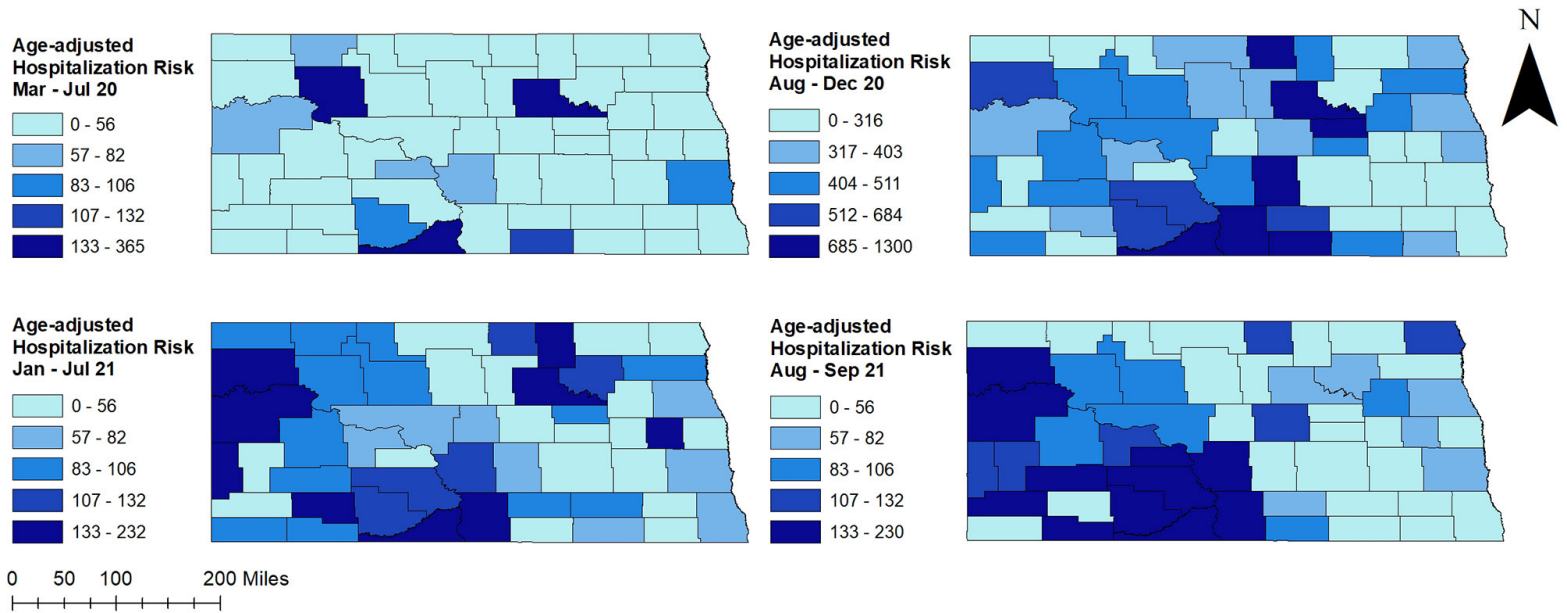
County-level unsmoothed age-adjusted COVID-19 hospitalization risks in North Dakota, March 2020–September 2021.

**Figure 3:**
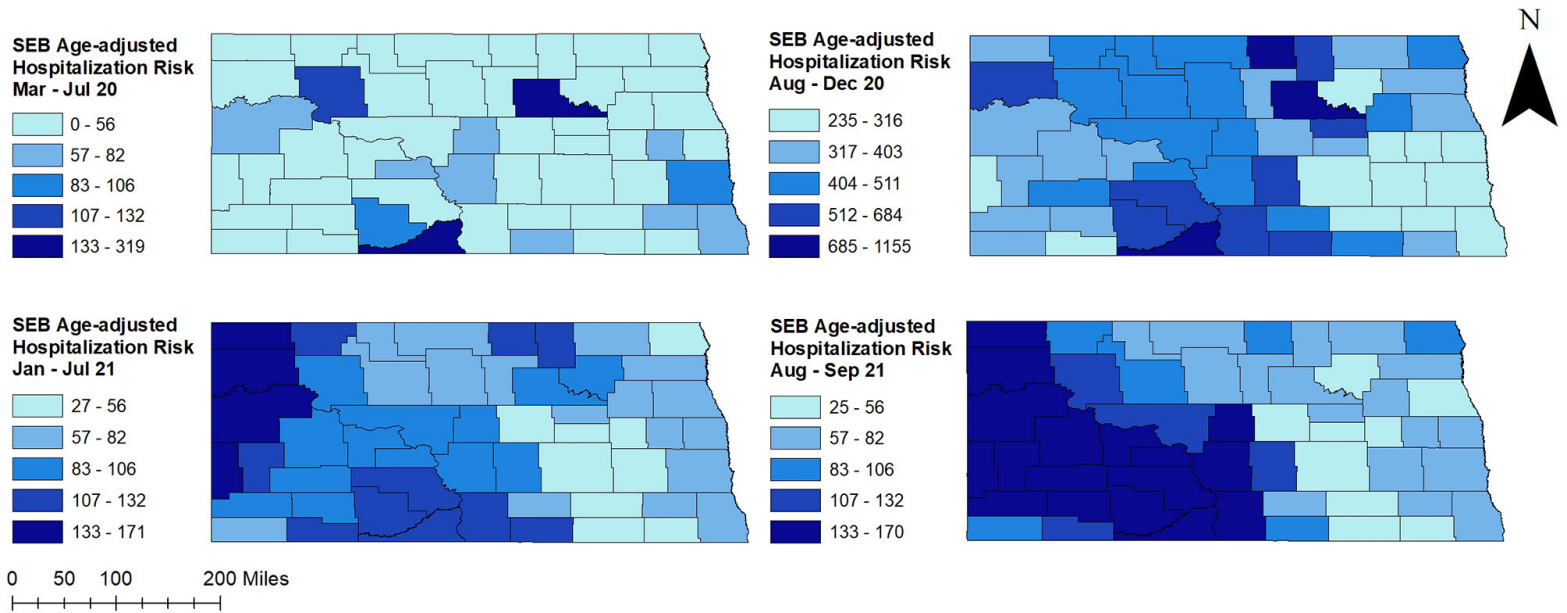
County-level spatial empirical Bayes (SEB) smoothed age-adjusted COVID-19 hospitalization risks in North Dakota, March 2020–September 2021.

**Figure 4:**
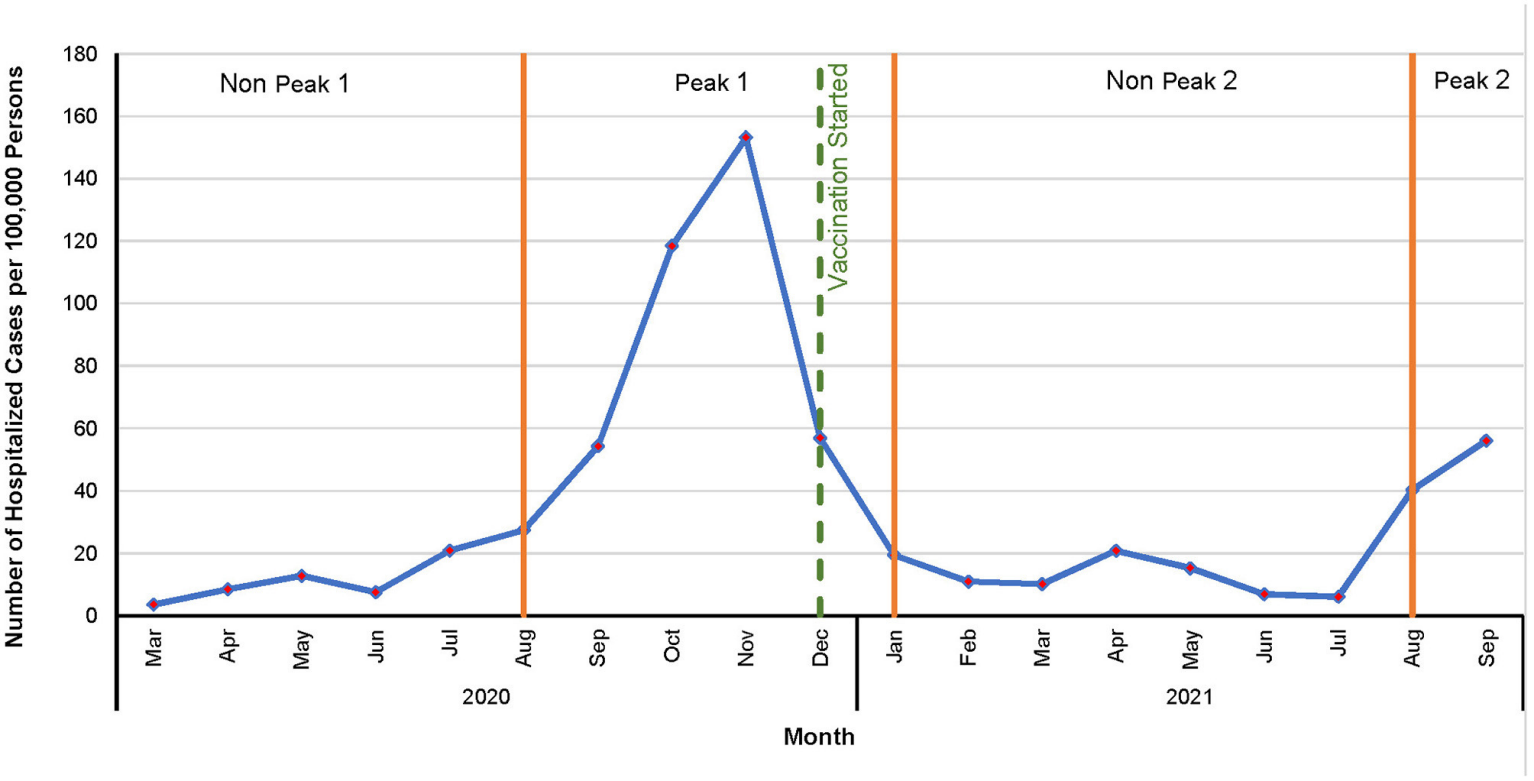
Temporal patterns of COVID-19 hospitalization risks in North Dakota, March 2020–September 2021.

There was a total of 4,938 COVID-19 hospitalizations during the study period. Age-adjusted COVID-19 hospitalization risks varied across counties ranging from 0 to 365 hospitalizations per 100,000 persons in March to July 2020 and January to September 2021. However, higher hospitalization risks were observed from August to December 2020 and ranged from 0 to 1,300 hospitalizations per 100,000 persons (Figures 2–4). At the beginning of the study period (March to July 2020), a few rural counties had high age-adjusted hospitalization risks (>56 hospitalizations per 100,000 persons), while almost half of the counties had high age-adjusted hospitalization risks during the rest of the study period (>316 hospitalizations per 100,000 persons in August to December 2020, >56 hospitalizations per 100,000 persons in January-September 2021). These included rural and urban counties (Figures 1–3). Counties in the western and central parts of the state tended to have consistently high age-adjusted hospitalization risks, while low age-adjusted hospitalization risks were observed in the east (Figures 2, 3).

Overall, COVID-19 hospitalization risks were relatively stable from January to July and spiked in the fall (Figure 4). A sharp increase in hospitalization risk was observed from August to November 2020, and a sharp decrease was observed thereafter in December 2020. A similar pattern of increase was observed in August 2021. Average hospitalization risk in peak period was significantly (*p* < 0.05) higher compared to non-peak period. The highest COVID-19 hospitalization risk was observed in November 2020 (153 hospitalizations per 100,000 persons) while the lowest was in March 2020 (4 hospitalizations per 100,000 persons) (Figure 4).

### 3.2. Clusters of high COVID-19 hospitalization risks

**Figure 5:**
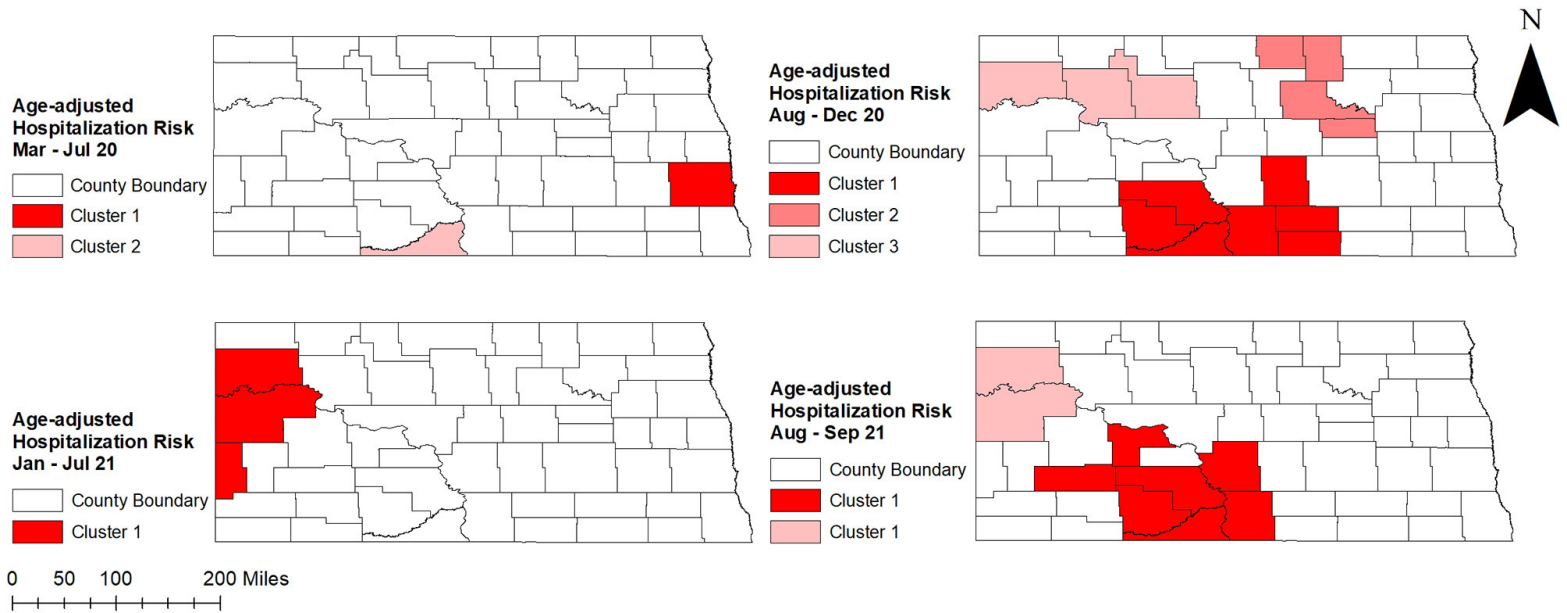
Spatial clusters of age-adjusted high COVID-19 hospitalization risks identified in North Dakota using Tango's flexible spatial scan statistics, March 2020–September 2021.

Figure 5 shows age-adjusted high COVID-19 hospitalization risk clusters identified in North Dakota using the Tango's FSSS. Consistent with high age-adjusted hospitalization risks observed in the western and central parts of the state, significant high hospitalization risk clusters were identified in these areas (Figures 2, 3, 5). There was increase in both the numbers of counties involved in the clusters and sizes of the populations affected in peak periods compared to non-peak periods (Table 1 and Figure 5).

**Table 1:** Spatial clusters of age-adjusted high COVID-19 hospitalization risks identified in North Dakota using Tango's flexible spatial scan statistics, March 2020–September 2021.

**Period**	**Cluster**	**Population**	**Observed cases**	**Expected cases**	**No. of counties**	**RR**	***p*-value**
March 2020–July 2020	Cluster 1	179,937	177	99	1	1.78	0.001
	Cluster 2	4,339	16	2	1	6.68	0.001
August 2020–December 2020	Cluster 1	47,783	326	186	7	1.76	0.001
	Cluster 2	25,750	202	100	4	2.02	0.001
	Cluster 3	115,354	573	448	3	1.28	0.001
January 2021–July 2021	Cluster 1	51,676	94	46	3	2.06	0.001
August 2021–September 2021	Cluster 1	144,905	288	167	7	1.72	0.001
	Cluster 2	49,880	85	47	2	1.79	0.001

RR, risk ratio.

**Figure 6:**
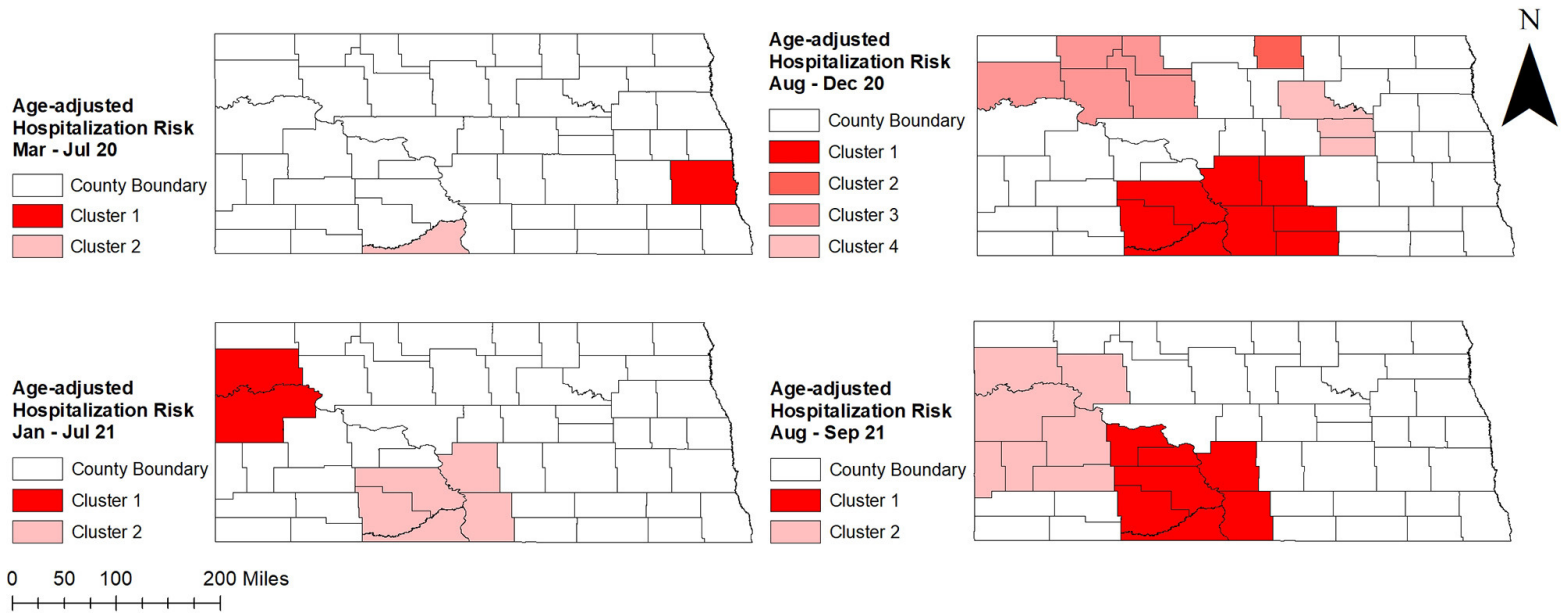
Spatial clusters of age-adjusted high COVID-19 hospitalization risks identified in North Dakota using Kulldorff's circular spatial scan statistics, March 2020–September 2021.

Two significant high hospitalization risk clusters were identified in peak periods of August to December 2020 and August to September 2021, one in the north-west and the other in the south-central part of the state. In addition, a high-risk cluster was identified in the north-central part of the state in August to December 2020 and included several counties (Rolette, Towner, Benson, Eddy) that were not part of any cluster identified in other peak and non-peak periods. On the other hand, a high hospitalization risk cluster involving only Cass county was identified in eastern North Dakota during the first non-peak period in March to July 2020. Another high hospitalization risk cluster (Sioux county) identified at the same time in the south-central part of the state had the highest hospitalization risk ratio (HRR = 6.68; *p* = 0.001) (Table 1 and Figure 5). During the second non-peak period in January to July 2021, a high-hospitalization risk cluster was identified in the western part of the state (Figure 5). The cluster identified in the second non-peak period included more counties and a larger population than that of the first non-peak period. Interestingly, Kulldorff's CSSS had almost similar findings to Tango's FSSS (Table 2; Figures 5, 6). However, an additional high-risk cluster was identified in Kulldorff's CSSS in the south-central part of the state during January–July 2021 (Figure 6).

**Table 2:** Spatial clusters of age-adjusted high COVID-19 hospitalization risks identified in North Dakota using Kulldorff's circular spatial scan statistics, March 2020–September 2021.

**Period**	**Cluster**	**Population**	**Observed cases**	**Expected cases**	**No. of counties**	**RR**	***p*-value**
March 2020–July 2020	Cluster 1	179,937	162	89	1	2.38	<0.001
	Cluster 2	4,339	13	2	1	7.16	<0.001
August 2020–December 2020	Cluster 1	143,292	833	632	8	1.43	<0.001
	Cluster 2	14,437	104	50	1	2.11	<0.001
	Cluster 3	119,883	516	419	5	1.28	<0.001
	Cluster 4	12,353	92	56	3	1.67	<0.001
January 2021–July 2021	Cluster 1	49,880	74	35	2	2.23	<0.001
	Cluster 2	136,546	172	128	5	1.46	0.003
August 2021–September 2021	Cluster 1	146,867	260	149	7	2.16	<0.001
	Cluster 2	98,436	137	84	7	1.77	<0.001

RR, risk ratio.

Williams county was consistently part of a high hospitalization risk cluster during peak and non-peak periods from August 2020 to the end of the study period (Figures 1, 5, 6). Several counties in the south-central part of the state (Morton, Grant, Sioux, Emmons) were part of high hospitalization risk clusters during both peak periods. However, some counties in central North Dakota (Rolette, Benson, Eddy, Ward) that were part of high-hospitalization risk clusters during the first peak period in August to December 2020 were not part of any cluster during the second peak period in August to September 2021. The opposite trend of transitioning from non-significant to statistically significant high hospitalization risk clusters was observed in McKenzie, Mercer, and Stark counties and these were mostly located in the western part of the state. These findings were consistent between Tango's FSSS and Kulldorff's CSSS methods (Figures 1, 5, 6). Burleigh county, where the state capital is located, was in part of high hospitalization risk cluster during the peak period but not during the non-peak period. During both non-peak periods, only Sioux county was consistently identified in high hospitalization risk clusters in the CSSS method (Figures 1, 6).

## 4. Discussion

This study investigated county-level geographic disparities and temporal changes of age-adjusted COVID-19 hospitalization risks in North Dakota. Although substantial disparities were reported in infectious disease morbidities and mortalities among North Dakotans, little is known about disparities of COVID-19 burden in North Dakota (25). The findings of the current study help to fill this gap and are useful in guiding evidence-based health planning and resource allocation in combating the COVID-19 problem. Since North Dakota is one of the most rural states, findings also help to understand the burden of COVID-19 in rural populations of the US (26).

The increase in age-adjusted hospitalization risk in fall 2020 and a decrease thereafter in early spring 2021 was consistent with the overall COVID-19 hospitalization trend observed in the US (27). The decline in COVID-19 hospitalization risks in spring 2021 could be due to the arrival of Food and Drug Administration (FDA) emergency authorized vaccines (28) and starting of vaccinations in December 2020 (29). In addition, there were restrictive policies such as social distancing, mandatory masking, and limited mobilities imposed during 2020 and early 2021, which resulted in low transmission of COVID-19 (30). However, the rise of new delta variant, relaxed COVID-19 restriction policies, and waning immunity could be responsible for the observed surge in July 2021 (30).

Although there was an overall increase in hospitalization risk starting from fall 2020 across counties in North Dakota, counties in the western and central parts of the state had higher hospitalization risks than the eastern part of the state. This is probably due to geographical differences in vaccination coverage. According to a report by NDHHS, percentages of populations having at least one dose of COVID-19 vaccine were substantially higher in counties of the eastern part of the state compared to the rest of the state (31). The reasons for the low vaccination rates in the western and central North Dakota could be lack of sufficient vaccine administration facilities and limited accessibility due to its rough terrain (32). Geographic barriers could also have prevented COVID-19 patients from getting supportive outpatient care which might have increased hospitalization risks in those areas. A Kaiser Family Foundation report stated that rural populations are less likely to get vaccine compared to urban populations due to lack of understanding of COVID-19 severity and vaccine effectiveness (13). However, a report published by the Office of the Assistant Secretary for Planning and Evaluation (ASPE) showed that there was a high percentage of unvaccinated individuals in North Dakota who were willing to get vaccinated despite living in rural areas (33).

High hospitalization risk clusters identified in south-central (Sioux) and east-central areas (Benson, Rolette) of the state could be due to racial distributions of populations of those areas. The majority of the population of those counties are American Indians (AI) (34, 35), who have the highest hospitalization risks among all minority populations in the US due to limited access to healthcare services and underlying health disparities (36, 37). In addition, the percentage of the population with health insurance and median household income among these populations were lower than the state average (38, 39). However, the percentage of the population that had received at least one dose of vaccine in 2021 was higher in Rolette and Benson counties than that of Sioux county (31). This could explain the fact that Sioux county was part of high hospitalization risk clusters in both peak periods, while Rolette and Benson counties were not included in any high hospitalization risk cluster in the second peak. Suffice it to say that vaccines may have been effective in reducing hospitalization risks in Rolette and Benson counties (40). Since all three counties have similar racial distribution, median income, and insurance coverage, the low vaccination rate in Sioux county could be due to structural barriers such as limited healthcare accessibility. A report published by ASPE supports this hypothesis showing that unvaccinated individuals of Sioux county were interested in getting vaccines (33). This implies that there were no behavior-related barriers to vaccination such as lack of trust or misperception (41, 42). Moreover, culturally inclusive programs implemented in north and east central counties might have played an important role in managing COVID-19 in those areas. A tribally managed CDC-guided comprehensive COVID-19 case management program was implemented among populations of Spirit Lake during the first peak period in late September 2020. Since this program was effective in controlling COVID-19 risks (9), similar programs could help to manage COVID-19 in other tribal areas and public health communities. Similar to AI people, non-Hispanic Black people are more likely to be hospitalized due to COVID-19 than non-Hispanic White people due to underlying health disparities (37, 43–45). Counties in the western part of the state have relatively high percentages of non-Hispanic Black populations which could have contributed to the high hospitalization risks observed in those areas. However, the eastern part of the state also has a relatively high percentage of non-Hispanic Black population but low hospitalization risk. The reason for this remains unclear. High vaccination coverage and better geographic accessibility to healthcare facilities might have reduced hospitalization risks in the eastern part of North Dakota.

### 4.1. Strengths and limitations

Use of two statistically rigorous spatial epidemiological approaches to investigate geographic disparities in age-adjusted COVID-19 hospitalization risk in North Dakota is a key strength of this study. Both FSSS and CSSS methods are robust, adjust for multiple comparisons, and are free of pre-selection bias. Additionally, the FSSS method identifies both circular and non-circular windows. Moreover, this is the first study investigating geographic disparities of COVID-19 hospitalization burden in North Dakota. Study findings are crucial for NDHHS targeting control efforts aimed at reducing disparities and improving health for all North Dakotans. However, this study is not without limitations. Confirmed COVID-19 hospitalized cases might be under-reported, especially at the beginning of the study period, because availability of COVID-19 testing varied across the state, and testing requirements changed over time.

## 5. Conclusion

This study has confirmed that geographic disparities in COVID-19 hospitalization risks exist in North Dakota. Specific attention is required to address counties with high hospitalization risks. Study findings are useful for guiding COVID-19 response geared at reducing disparities and improving COVID-19 outcomes.

## Data availability statement

The original contributions presented in the study are included in the article/Supplementary material, further inquiries can be directed to the corresponding author.

## Ethics statement

This study was reviewed and approved by the University of Tennessee Institutional Review Board (IRB). The IRB number is UTK IRB-22-07032-XM. All study methods were carried out in accordance with relevant guidelines and regulations. The study used data provided by the North Dakota Department of Health and Human Services. The identity of human subjects cannot be ascertained directly or through identifiers linked to the subjects. The investigators did not contact and re-identify subjects. Since the study used secondary data, no human participants were recruited by the investigators, and therefore, the University of Tennessee IRB granted a waiver for consent to participate.

## Author contributions

MK and AO conceptualized research idea and analyzed data. MK, ND, and AO wrote the manuscript. MS and GN were involved in manuscript review. All authors read and approved the final manuscript.
